# Benign Paroxysmal Positional Vertigo without nystagmus: diagnosis and treatment

**DOI:** 10.1590/S1808-86942011000600018

**Published:** 2015-10-19

**Authors:** Gabriella Assumpção Alvarenga, Maria Alves Barbosa, Celmo Celeno Porto

**Affiliations:** 1 MSc student in Health Sciences - Federal University of Goiás, Professor at the Pontfícia Universidade Católica de Goiás; 2 PhD. Adviser at the Graduate Program in Health Sciences - Universidade Federal de Goiás, Full Professor - Nursing School - Universidade Federal de Goiás; 3 PhD. Adviser at the Graduate Program in Health Sciences - Universidade Federal de Goiás, Emeritus Professor - Medical School - Universidade Federal de Goiás

**Keywords:** diagnosis, therapeutical approaches, vertigo

## Abstract

**Abstract:**

Nystagmus tests to diagnose BPPV are still relevant in the clinical evaluation of BPPV. However, in everyday practice, there are cases of vertigo caused by head movements, which do not follow this sign in the Dix-Hallpike maneuver and the turn test.

**Aim:**

To characterize BPPV without nystagmus and treatment for it.

**Materials and methods:**

A non-systematic review of diagnosis and treatment of benign paroxysmal positional vertigo (BPPV) without nystagmus in the PubMed, SciELO, Cochrane, BIREME, LILACS and MEDLINE databases in the years between 2001 and 2009.

**Results:**

We found nine papers dealing with BPPV without nystagmus, whose diagnoses were based solely on clinical history and physical examination. The treatment of BPPV without nystagmus was made by Epley maneuvers, Sémont, modified releasing for posterior semicircular canal and Brandt-Daroff exercises.

**Conclusion:**

From 50% to 97.1% of the patients with BPPV without nystagmus had symptom remission, while patients with BPPV with nystagmus with symptom remission ranged from 76% to 100%. These differences may not be significant, which points to the need for more studies on BPPV without nystagmus.

## INTRODUCTION

Benign Paroxysmal Positional Vertigo (BPPV) is one of the most frequent vestibular disorders. Its incidence varies between 11 and 64 cases per 100 thousand inhabitants^1^. It predominates in the age range between 50 and 55 years in idiopathic cases^2^ and it is rare in childhood^3^. It is more frequently seen at older ages because of the degeneration of statoconia, arising from demineralization, shown by means of histopathology studies^4^.

According to Weider et al.^5^, the first to describe BPPV was Busch, in 1882. After the first description, the papers considered important were published by these authors: Adler in 1897 and Báràny^6^ in 1921.

As far as etiopathogeny is concerned, Schuknecht^7^ and Schuknecht & Ruby^8^ called cupulolithiasis the deposit of these particles in the posterior semicircular canal. Hall et al.^9^ suggested that these particles would be floating, which is called canalithiasis. Gans^10^ stated that everyone has an amount of free statoconia in their semicircular canals. Nonetheless, the body is normally able to totally absorb the calcium within a few hours or days, without triggering symptoms. These would be triggered when the body metabolism has difficulties in absorbing calcium. In the presence of these free calcium carbonate particles in the semicircular canals coming from the fractioning of the otoliths in the utricular macula and in enough quantity to activate the nerve endings, vertigo is triggered during head movement, thus characterizing BPPV.

The most often involved semicircular canal in the BPPV is the posterior^11^, nonetheless, there may be otolith deposits in the lateral and anterior semicircular canals^12^.

Among the causes associated with BPPV, the most common are head injury (17%) and vestibular neuritis (15%). Other causes include vertebrobasilar ischemia, labyrinthitis and surgical complications from middle ear intervention and after prolonged rest. Nonetheless, most of the cases seem to be idiopatic^2^.

For diagnostic purposes, the positioning nystagmus investigation enables the localization of the side and that of the damaged canal and the distinction between canalithiasis and cupulolithiasis, being important to guide the most indicated rehabilitation exercises for each case, a fundamental part of treatment^12^.

Dix & Hallpike^13^ were responsible for establishing the objective criteria for BPPV diagnosis. They described a maneuver which helps evaluate vertigo and positioning nystagmus and proposed the name of BPPV for this disorder which included this symptom and sign. These authors described that upon the maneuver, nystagmus would be triggered after a latency time, disappearing after the maneuver is repeated two or three times; nonetheless, the diagnosis of BPPV was only considered in the presence of nystagmus.

The Dix and Hallpike maneuver have a positive predictive value of 83% and negative predictive value of 52% for the diagnosis of posterior and anterior semicircular canal BPPV, and a common mistake is not to perform it in patients complaining of vertigo or dizziness^14^,^15^^-^^16^. The Brandt-Daroff test, or the turn test is used to look for lateral canal positional nystagmus^12^,^17^. In general, it is not recommended to order image complementary exams, vestibular tests, or both in patients clinically diagnosed with BPPV, unless it is uncertain or when there are other signs and symptoms in the BPPV tests^17^.

Nystagmus in these cases is considered important to characterize the BPPV until current days. Nonetheless, in the clinical practice, there are cases of vertigo caused by movements such as: laying down, turning from one side to the other in bed, fast head movements horizontally and bending over, without nystagmus in the Dix -Hallpike maneuaver^18^,^19^.

BPPV studies^2,^^20^, ^21^, ^22^, two systematic reviews among them^2^,^20^, approached the BPPV treatment without mentioning the diagnostic difficulty in the absence of nystagmus. As reported by Silveira & Munaro^23^, there is a shortage of studies on this subject. On BPPV studies^2^,^20^; in general, the patients who did not have nystagmus are taken off the study, especially when the study aims at proving treatment when the lack of this signal characterizes the study outcome.

Treatment with exercises and vestibular rehabilitation repositioning maneuvers depend on the identification of the damaged canal and are not specific for each one of them^12^. Upon nystagmus and detecting the semicircular canal involved, the canalith repositioning maneuver has been proven efficient (especially that of Epley for the posterior semicircular canal)^1^,^20^. Nonetheless, in the absence of nystagmus, would it be possible to diagnose and treat BPPV?

Given the aforementioned, added to the scarce publications on BPPV without nystagmus, also called subjective or atypical, this non-systematic review is fully justified and its goal is to characterize the BPPV without nystagmus, as well as the treatment approach in such situations.

## METHODOLOGY

We searched for papers in the following databases: MEDLINE, BIREME, SCIELO, LILACS, PUBMED starting from the keywords which characterized the topic: BPPV, lack of nystagmus, diagnosis and treatment, in Portuguese, English and German.

The selection criteria for studies were: published between 2001 and 2009; clinical studies with adults and literature reviews with emphasis in the diagnosis and treatment of BPPV without nystagmus. Added to this review is the summary, in English of a paper in Chinese^24^, available at PUBMED. One of the papers corresponding to the criteria of this study^25^ was not found.

## RESULTS

Of the ten listed papers, we found nine^18^,^19,^^23^, ^24^, ^25^, ^26^, ^27^, ^28^, ^29^, ^30^ (Chart 1).

**Chart 1:** Papers discussing BPPV without nystagmus, published between 2001 and 2009.

Author/Rev./Year	Study Type	Series	Results	Considerations and comments
Tirelli et al.^18^/Laryngoscope/ 2001	Clinical prospective	43 patients with BPPV without nystagmus, 24 (53.5%) with vertigo, 3 (7%) with nausea, 10 (23.2%) with vertigo and nausea in the positional tests (Dix- Hallpike and Sémont with Frenzel goggles). They were treated by the modified PSCC (posterior semicircular canal) repositioning maneuver. The reassessment was carried out after 5 days.	Complete recovery: 26 (60.46%) patients; 14 (32.56%) partial recovery and 3 (6.9%) did not perceive changes in the symptoms. None of the patients perceive a worsening in their condition. All the 17 (39.46%) patients who did not experience symptom remission were submitted to a new diagnostic investigation, obtaining diagnostics which were different from BPPV.	The maneuver is of low cost and it is not inconvenient to the patient. In the case of treatment failure, new tests were carried out with the goal of looking for other causes for vertigo, excluding BPPV.
Haynes, DS et al./Laryngoscope/ 200^2^,^19^	Clinical Prospective Comparative	127(78.4%) patients with BPPV and nystagmus and 35(21.6%) with BPPV without nystagmus, detected in the Dix-Hallpike maneuver without the Frenzel goggles, were submitted to the Sémont treatment maneuver, and reassessed 3 weeks later.	97(76%) patients with BPPV and nystagmus had complete symptom remission, 19 (15%) patients reported improvement. Among the patients with BPPV without nystagmus, symptom remission happened to 22 (63%) patients and 8 (23%) reported improvements. There were not statistically significant differences in the treatment of BPPV with and without nystagmus	The Sémont maneuver proved efficient both in BPPV with nystagmus as in that without nystagmus, with a 13% difference in symptoms improvement. It is a low cost procedure, usually well tolerated by patients.
Ganança MM/ Acta AWHO/200^2^,^25^	Paper not found, *apud* Koga et al.^26^ (2004)			
Koga et al.^26^ / Rev. CEFAC/2004	Cross-sectional Descriptive Observationa	l167 patients with vertigo and/ or dizziness were assessed by means of vector-electronystagmography and the Dix Hallpike test with the Frenzel goggles, with the goal of checking the prevalence of dizziness and/or vertigo associated with head movement and the main alterations found in the vestibular test.	Of the 167 patients, 68 (40.8%). Complained of dizziness and/ or vertigo associated with a change in head position, which was characterized as BPPV, even in the absence of nystagmus. Only 7 (10.3%) had positional or positioning nystagmus visible with the Frenzel goggles.	The authors characterized BPPV without nystagmus in the Dix Hallpike test using the Frenzel goggles, in patients with dizziness and/or vertigo in this positioning.
Ganança MM et al.^27^/ Acta ORL/ 2005	Review paper	The goal of this paper was to present a review of the main diagnostic and treatment aspects associated with BPPV. It reinforces the use of Frenzel goggles (of 20 dioptries) or the videonystagmography to study nystagmus type and direction, which according to the authors is difficult upon simple observation.	They assessed 17 papers published between 1990 and 2002.	They considered BPPV in the presence of vertigo without nystagmus detected in the Dix Hallpike test and stated that nystagmus was present in 50% of the cases. This lack of nystagmus is attributed to habituation because of regular daily head movements. They report that the BPPV treatment in the absence of nystagmus is not different from the treatment with nystagmus, identifying the labyrinth involved by means of vertigo upon change in head position.
Zhonghua et al.^24^/ Paper published in Chinese/2007/Abstract in English.	Comparative retrospective clinical analysis	The goal was to assess the clinical and therapeutic characteristics of BPPV comparing BPPV without nystagmus (12 patients) with BPPV with nystagmus (24 patients). BPPV was characterized by the Dix-Hallpike test.	Complete symptom remission was noticed in 11(97.1%) patients with BPPV without nystagmus and in 19(79.2%) patients with nystagmus. Treatment was carried out by the use of a repositioning maneuver (not specified in the paper's abstract).	Original paper in Chinese. BPPV treatment was better in patients without nystagmus when compared to those with it. In the abstract there is no reference as to the use of Frenzel goggles.802
Anagnostou E et al.^28^/Original paper in German/HNO 3/2007	Retrospective clinical analysis	70 patients complaining of dizziness were analyzed. Of these, 37 (54.1%) had a typical history of BPPV with nystagmus and 33 (48.6%) had typical history without nystagmus which was confirmed by the Dix-Hallpike test and the lateralization maneuver.	Thirty seven (54.1%) patients had a typical history with nystagmus, 30 (83%) were treated with Epley and 7 (17%) with Sémont, becoming symptom-free. Thirty three (48.6%) had typical history without nystagmus and were treated with Brandt-Daroff exercises; 50 % of them did the exercises at home and had complete symptom remission.	The patients were contacted by phone one year after treatment, at the time the retrospective study was being carried out, and questions were made by means of a structured questionnaire. They noticed that even with a normal neurotological exam, a typical medical history of BPPV, even without overt nystagmus upon positioning, diagnosis and treatment can be carried out, avoiding unnecessary complementary tests.
Johkura K; Momoo, T; Kuroiwa, Y^29^/ J Neurosurg Psychiatry/ 2008	Comparative Clinical Prospective	155 patients complaining of dizziness were part of the control group and 200 patients complaining of chronic dizziness (not accompanied by hearing loss, tinnitus and changes in the MRI), were assessed by means of a camera with infra-red lighting with the Frenzel goggles and video-oculography (the diagnostic test was not specified).	A subtle nystagmus matching that of horizontal semicircular canal BPPV was seen in 98 of the 200 patients with chronic dizziness and in 155 patients without complaints of dizziness. The typical history of BPPV was present in 69 (34.5%) of the patients complaining of dizziness, and in 18 (11.6%) in the control group. The patients with HSCC BPPV (49), detected by means of the medical history and the presence of nystagmus were treated by Brandt-Daroff home exercises for one year. There was a trend towards better symptom remission (*p*=0.0529) among the 49 (24.5%) patients who underwent the exercises when compared to the 77(13%) who did not do it.	The precise mechanism behind the subtle nystagmus persistence and the BPPV chronicity are unknown. HSCC BPPV seems to be a relatively common cause of dizziness in the elderly. There was no report on the one year follow up of the patients submitted to the Brandt-Daroff exercises.
Munaro G &Silveira AF^23^/Rev. CEFAC/ 2009	Observational Comparative Cross-sectional	86 patients with clinical history of BPPV who were assessed by means of the positioning tests (Dix-Hallpike and the *roll maneuver*) and vector-electronystagmography.	45 (49.45%) had nystagmus and 41 (45.04%) did not have it. The complaint of vertigo was common in both groups. Disease duration and the occurrence of associated diseases were divergent, being higher in patients with BPPV without nystagmus.	BPPV without nystagmus was called atypical and with nystagmus was considered typical.
Caldas et al. Rev Bras Otorrinol.; 200^9^,^30^	Retrospective Series	They analyzed the charts from 1271 consecutive patients examined in the past 6 years with BPPV by means of the Dix-Hallpike maneuver and the Frenzel goggles.	BPPV had prevalence in the age range between 41 and 60 years. 473 (42.2%), females 798 (62.8%), nystagmus and positioning vertigo in 1033 (81.3%). Cure or improvement by means of the particle repositioning maneuver (77.9%); and the possibility of recurrence (21.8%, in one year of follow up). BPPV with vertigo and without positioning nystagmus happened in 238 patients (18.7%).	As far as clinical evolution is concerned, 990 (77.9%) patients became asymptomatic or improved after the first treatment done by means of the repositioning maneuver. There was no specification as to nystagmus absence or presence as well as the type of repositioning maneuver used. The patients were followed up by one year, without a description of how this process was executed with the treated patients, BPPV recurrence was found in 277 (21.8%) cases; however, the paper is not specific as to their clinical presentation. Four patients (3.2%) maintained symptomatic and there was no characterization of the nystagmus presence or absence in this situation.

## DISCUSSION

As we can see on Chart 1, in the studies with BPPV without nystagmus, one was a bibliography review study^27^, two were observational cross-sectional studies^23^,^26^, three were retrospective clinical analyses^24^,^28^,^30^ - one comparative^24^ and three prospective clinical analyses^18^,^19^,^29^, two of which were comparative^19^,^29^.

BPPV without nystagmus is characterized by the clinical exam in which the patients complaining of brief BPPV spells without nystagmus and/or nausea associated with changes in head position did not have positional and/ positioning nystagmus^18^,^19,^^23^, ^24^, ^25^, ^26^, ^27^, ^28^, ^29^, ^30^.

Caovilla & Ganança^31^ state that the possible results from the Dix-Hallpike test in BPPV with and without nystagmus are: positive objective, when there is nystagmus associated with vertigo, positive subjective when there is only vertigo and negative in the absence of nystagmus and vertigo.

We found three probable explanations for the absence of dizziness and positioning nystagmus in head movement which would enable symptom and ocular phenomenon elimination at that time. The patients could have minimum calcium carbonate particles stuck to the cupule or floating in the affected semicircular canal, enough to cause nausea and/or vertigo, but not enough to cause nystagmus. In this situation, the affected labyrinth would be the one on the same side of the maneuver from which the patient reported dizziness seating down. Before treating the patient, the maneuver can be negative for BPPV in a first assessment and positive in another one, on the same day or in a different day. Many BPPV cases did not have positioning nystagmus or dizziness at the time of the maneuver, which does not rule out the diagnostic maneauver^18^,^19^,^27^.

Another explanation for the BPPV without nystagmus was proposed by Johkura, Momoo & Kuroiwa^29^. They perceived that among elderly citizens with chronic dizziness of unknown cause, without nystagmus in the conventional assessment using Frenzel goggles, diagnosis is very difficult. After investigating 200 elderly with dizziness, in whom they used an infrared camera and video-oculography, they found a faint positional ageotropic horizontal nystagmus, compatible with horizontal semicircular canal (HSCC) BPPV in 98 patients. It is also stressed that the mechanism of this mild nystagmus in the elderly is unknown, and they are also not eligible to make up for the balance disorder caused by this BPPV. These authors consider that the prevalence of this mild nystagmus is high and its history matches that of BPPV in the elderly, suggesting that the HSCC BPPV is one relatively common cause of chronic dizziness considered of unknown cause in the elderly.

Gans^10^ presents a third explanation based on a change in the calcium metabolism and the consequent non-absorption of free otoliths, which would increase their quantity in the semicircular canals and enable the triggering of vertigo upon head movement.

They did not present nystagmus in the diagnosis of BPPV in cross-sectional studies^23^,^26^ and prospective^18^,^19^,^29^ and retrospective^24^,^28^,^30^ cohorts in 9.6% to 89.7% of the patients with a mean value of 42% of the patients -, which is similar to the one found in the Ganança et al.^27^ bibliographic review, which considered nystagmus present in 50% of the patients.

Dix-Hallpike was the one most used in the studies^18^,^19^,^23^,^24,^^26^, ^27^, ^28^,^30^, with Frenzel goggles^18^,^26^,^27^,^30^ for the diagnosis of BPPV, with or without nystagmus and its characteristics. It is very difficult to recognize the positioning nystagmus type and direction upon simple observation, because this ocular phenomenon is mild and short lasted. The use of the Frenzel goggles (20 dioptries) or videonystagmography (VNG) enables the proper identification of the positioning nystagmus, allowing the pinpointing of the semicircular canal involved in the BPPV. The Frenzel goggles and the VNG rule out the inhibiting effect of the eye fixation on the vertical and horizontal nystagmus, this happens because the rotational nystagmus is not inhibited by eye fixation.

The treatment of BPPV without nystagmus was carried out by means of the Epley^28^ and Sémont^19^,^28^ maneuvers, the Brandt-Daroff^28^,^29^ exercises and the Modified Posterior Semicircular Canal Maneauver^18^. Nonetheless, two studies did not mention the maneuvers utilized^24^,^30^.

As far as treatment for BPPV patients without nystagmus is concerned^18^,^19^,^24^,^28^, 50 to 97.1% of the patients (mean value of 67.64%) had remission.

In the studies^19^,^24^,^28^ which compared treatment results from patients with and without nystagmus, symptom remission was 17% greater among patients with nystagmus. Haynes et al.^19^ did not find a significant difference (13%) among patients with and without nystagmus. On the other hand, Zhonghua et al.^24^ stated that the patients without nystagmus had a significantly higher improvement when compared to the patients who had BPPV with nystagmus (17.9%).

In the three prospective studies, there were no similarities in the follow up of these patients. One reassessed the patients after 5 days^18^, another reassessed them after 3 weeks^19^ and the third^29^, after one year.

Most of the BPPV cases, with or without nystagmus responded favorably to vestibular rehabilitation physical therapy procedures. Ganança et al.^27^ stated that vertigo upon head position change enables the identification of the labyrinth involved in the BPPV without nystagmus. Failures can happen because of the movement of crystals to another semicircular canal, creating another BPPV variant.

## CONCLUSION

BPPV without nystagmus is characterized by vertigo and/or nausea in the absence of nystagmus, especially in the Dix-Hallpike and in the Sémont, Brandt-Daroff tests or in the turn test or lateralization maneuver. Frenzel goggles with infrared camera were not used in all the patients, but they may be useful.

The treatment of BPPV without nystagmus may be carried out based on the typical history of BPPV and signs found upon physical examination, with vertigo. One should treat the side on which the signs were triggered by means of the Epley and Sémont maneuvers and the Brandt-Daroff exercises, or even, by means of the modified freeing maneuver for the posterior semicircular canal.

Symptom remission among patients with BPPV without nystagmus who were treated was of 67.64%, with a subtle difference for patients with nystagmus (13% to 17%), which suggests the need for BPPV treatment, even in patients without nystagmus.
